# Coinvasion by the ladybird *Harmonia axyridis* (Coleoptera: Coccinellidae) and its parasites, *Hesperomyces virescens* (Ascomycota: Laboulbeniales) and *Parasitylenchus bifurcatus* (Nematoda: Tylenchida, Allantonematidae), in the Caucasus

**DOI:** 10.1371/journal.pone.0202841

**Published:** 2018-11-29

**Authors:** Marina J. Orlova-Bienkowskaja, Sergei E. Spiridonov, Natalia N. Butorina, Andrzej O. Bieńkowski

**Affiliations:** A. N. Severtsov Institute of Ecology and Evolution, Russian Academy of Sciences, Moscow, Russia; Lund University, SWEDEN

## Abstract

The study of parasites in recently established populations of invasive species can shed light on the sources of invasion and possible indirect interactions between the alien species and native ones. We studied parasites of the global invader *Harmonia axyridis* (Coleoptera: Coccinellidae) in the Caucasus. In 2012, the first established population of *Ha*. *axyridis* was recorded in the Caucasus in Sochi (south of European Russia, Black Sea coast). By 2018, the ladybird had spread to a vast area: Armenia, Georgia and south Russia (Adygea, the Krasnodar territory, the Stavropol territory, Dagestan, Kabardino-Balkaria and North Ossetia). The examination of 213 adults collected in Sochi in 2018 showed that 53% were infested with *Hesperomyces virescens* fungi (Ascomycota: Laboulbeniales) and that 8% were infested with *Parasitylenchus bifurcatus* nematodes (Nematoda: Tylenchida, Allantonematidae). The examined *Ha*. *axyridis* specimens were free of the parasitic mite *Coccipolipus hippodamiae*. An analysis of the phylogenetic relationships of *P*. *bifurcatus* based on 18S rDNA confirmed the morphological identification of this species. *Hesperomyces virescens* and *P*. *bifurcatus* were first recorded in the Caucasus and Russia, although they are rather widespread in Europe. This likely indicates that they appeared as a result of coinvasion with their host because the populations of *Ha*. *axyridis*, *He*. *virescens* and *P*. *bifurcatus* in the Caucasus are isolated from the main parts of the ranges of these species in Europe. The nearest localities of *Ha*. *axyridis* is on another shore of the Black Sea, and the nearest localities of *He*. *virescens* and *P*. *bifurcatus* are more than 1000 km from the Caucasus. It is impossible to determine whether the first founders of the Caucasian population were infested with the parasites or whether the parasites were introduced by specimens of *Ha*. *axyridis* that arrived later from Europe. *Harmonia axyridis* was released in the region for pest control, but laboratory cultures are always free of *He*. *virescens* and *P*. *bifurcatus*. Therefore, the detection of *He*. *virescens* and *P*. *bifurcatus* indicates that the population of *Ha*. *axyridis* in the Caucasus could not have derived exclusively from released specimens. We did not find *He*. *virescens* on 400 specimens of 29 other ladybird species collected from the same localities as *Ha*. *axyridis* in the Caucasus. No reliable correlation between infestation by *He*. *virescens* and that by *P*. *bifurcatus* has been found. In addition to these two parasites, an unidentified species of the order Mermithida was recorded. This is the first documented case of *Ha*. *axyridis* infestation by a parasitic nematode of this order in nature.

## Introduction

Despite a large body of work on invasion ecology, the interactions between invasive species and their natural enemies, particularly parasites, are poorly studied [1]. The study of parasites of alien species in young, recently established populations is of great importance to understand the routes of invasion [2]. In addition, some parasites of alien species can affect native species, and vice versa [3]. Therefore, the study of parasites might reveal possible indirect interactions between an alien species and native ones and some reasons for its invasion success. The aim of our investigation was to determine which parasites occur on the harlequin ladybird, *Harmonia axyridis* (Pallas) (Coleoptera: Coccinellidae), in the Caucasus, which has been recently invaded by this species.

*Harmonia axyridis*, which is native to East Asia, has been widely introduced for the biological control of agricultural pests and is established almost all over the world (see the detailed description of the native range [4] and the overview of the global invasion [5]). It became established in Western Europe in the late 1990s and then rapidly expanded its range. Outbreaks of *Ha*. *axyridis* in some regions have caused a number of negative ecological consequences, including declines in native ladybird species [6]. By approximately 2010, the expansion of the European range to the east had reached Russia and adjacent regions [7]. The reasons for the great invasion success of this species and the decline of native ladybirds has attracted the attention of hundreds of scientists (see the review by Roy et al. [5]). Recent studies have shown that some symbionts of this global invader (parasites and microorganisms) have contributed to its success and have sometimes even become "biological weapons" against its competitors—native ladybirds [1, 3, 8]. Another factor that could have contributed to the overcoming of ecological competition is that *Ha*. *axyridis* in its invasive range is significantly less parasitized by the wasp *Dinocampus coccinellae* than native ladybirds [9].

**Fig 1:**
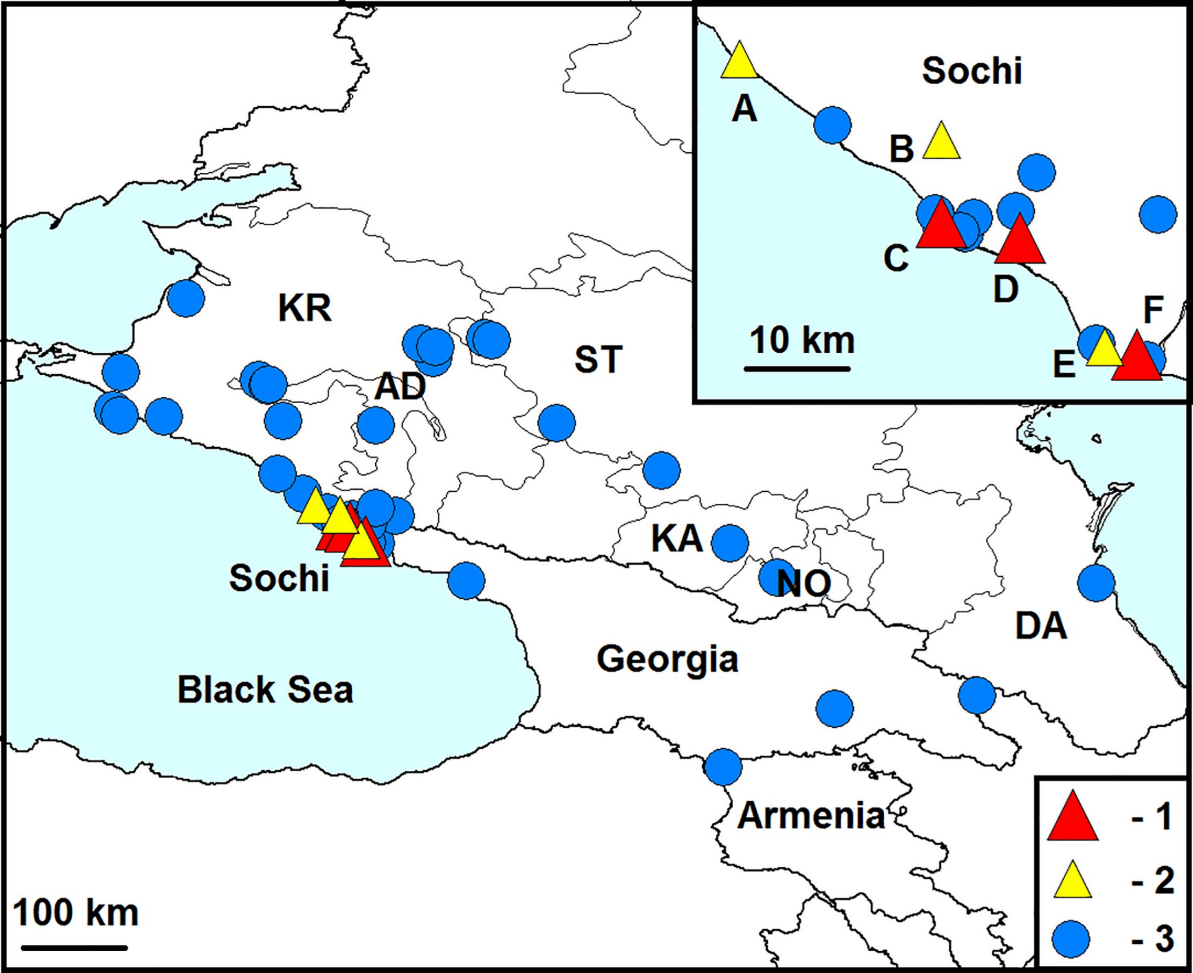
Known localities of *Harmonia axyridis* and its parasites in the Caucasus. 1 –localities of infestation of *Ha*. *axyridis* with *Hesperomyces virescens* and *Parasitylenchus bifurcatus*, 2 –localities of infestation of *Ha*. *axyridis* with *He*. *virescens*, 3 –other localities inhabited by *Ha*. *axyridis*. Regions of Russia: AD–Adygea, DA–Dagestan, KA–Kabardino-Balkaria, KR–the Krasnodar territory, NO–North Ossetia, ST–the Stavropol territory. Localities where the parasites were detected: A–Golovinka, B–Razbityj Kotel, C–Central District, D–Agur, E–Adler, F–Veseloe. A description of the localities and sources of information are provided in the supporting information (S1 Appendix). The map was prepared using the program DIVA-GIS (http://diva-gis.org/download) [18]. These maps were based on GADM maps under a CC BY license with permission from Robert Hijmans, owner of GADM (original copyright 2001).

*Harmonia axyridis* has been widely used for the biological control of Aphididae and other pests in the Caucasus since 1927. Specifically, in the 1980s, more than 107,000 specimens brought from the Far East were released in Georgia [10]. However, despite these massive releases, *Ha*. *axyridis* did not become established before the 21st century. The first established population in the region was recorded in Sochi in 2012 [11, 12]. Then, *Ha*. *axyridis* quickly spread and became common across the Black Sea coast of the Caucasus and adjacent regions. By 2018, it has been recorded in Armenia, Georgia and south Russia: Adygea, the Krasnodar territory, the Stavropol territory, the Rostov region, Dagestan, Kabardino-Balkaria and North Ossetia (Fig 1) [13, 14, 15]. The releases of *Ha*. *axyridis* continued at least to 2010 [16]. Therefore, it is unclear whether the population in the Caucasus originated from the released specimens or appeared a result of an expansion of the invasive European range of the species [12, 15, 17].

The establishment of *Ha*. *axyridis* in the Caucasus has led to three main questions:

1. What are the sources of the invasion of *Ha*. *axyridis* in the Caucasus?
2. Why is *Ha*. *axyridis* currently established in the region although it previously failed to establish despite massive releases?
3. How will *Ha*. *axyridis* affect native ladybird species?

Although our study of parasites cannot directly answer these difficult questions, it can likely shed some light on them.

We restrict usage of the term ‘‘parasites” to those organisms living at the expense of a single host that are also multicellular (in contrast to pathogenic microorganisms) and do not directly cause the death of the host (in contrast to parasitoids) [1]. Three species of parasites have been recorded to infest *Ha*. *axyridis* in nature around the world: *Hesperomyces virescens* Thaxt. fungi, *Coccipolipus hippodamiae* (McDaniel and Morrill) mites, and *Parasitylenchus bifurcatus* Poinar and Steenberg nematodes [1].

*Hesperomyces virescens* (Ascomycota: Laboulbeniales) is an obligate ectoparasite that has been reported to infect the adults of over 30 ladybird species (Coleoptera: Coccinellidae) in Europe, Asia, North America, South America and Africa [1]. The *Ha*. *axyridis*–*He*. *virescens* association has been reported in Austria, Belgium, the Czech Republic, Croatia, Germany, France, Hungary, the Netherlands, Poland, Slovakia, the UK, the USA, Canada, Argentina, Ecuador, South Africa, China [1], Bulgaria, Greece [19], and Mexico [20]. *Hesperomyces virescens* has not previously been detected in Russia or in the Caucasus [1]. The native range of this fungus is unknown. It was originally assumed that it could have originated in North America [21]. However, later, *He*. *virescens* was also found on specimens of *Ha*. *axyridis* collected in China in the 1930s [22]. Its entire life cycle takes place on the integument of adult ladybirds. The sticky spores have a short life span and are exclusively spread by the activities of the host, and transmission takes place during direct contact between individuals (mating or overwintering in aggregations) [23].

*Coccipolipus hippodamiae* (Acarina: Podapolipidae) is an ectoparasitic mite species. All life stages live on the underside of the elytra of adult ladybirds and feed on the hemolymph. This species is reported to affect five Coccinellidae species in North America, Africa and Europe. Transmission also takes place during mating or overwintering in aggregations [1].

*Parasitylenchus bifurcatus* (Nematoda: Allantonematidae) is an obligate endoparasite. Its only known host is *Ha*. *axyridis*. Fertilized infective females enter adult ladybirds through the spiracles or thin parts of the integument. They develop into females of the first parasitic generation, producing eggs, from which the females of the second parasitic generation develop. The latter give birth to juvenile males and females, which mature and mate inside the body of the host. Then, new infective females appear and leave the host. The mechanism of transmission is unknown [24]. *Parasitylenchus bifurcatus* was originally described in Denmark [24], and it has also been reported in the Netherlands [25], the Czech Republic, Poland [1], the USA and Slovenia [26]. There have been no documented records of the infestation of *Ha*. *axyridis* by nematodes belonging to the order Mermithida in nature.

No parasites of *Ha*. *axyridis* in the Caucasus or European Russia have been recorded [1]. We have found *He*. *virescens*, *P*. *bifurcatus* and an unidentified species belonging to the order Mermithida on *Ha*. *axyridis* in the region.

## Materials and methods

### Collection of *Ha*. *axyridis*

Adult *Ha*. *axyridis* (213 specimens) were collected in the city of Sochi (southern European Russia, the Krasnodar territory, Black Sea coast of the Caucasus) in April 17–May 15, 2018. The sites of collection were Veseloe (43.41, 39.98), Central District (43.58, 39.73), Adler (43.42, 39.94), the valley of the Agur River (43.56, 39.83), Golovinka (43.79, 39.47), and Razbityj Kotel (43.69, 39.73). The beetles were collected by shaking the branches of different trees and shrubs and sweep-netting grasses. It is likely that all these specimens had overwintered, since larvae were first detected on May 1 and pupae on May 11. No specimens of the young generation with soft elytra were detected. All beetles were placed in plastic containers and kept alive at a temperature of approximately +4°C.

No specific permission was required for collection of *Ha*. *axyridis*, since collection sites were not situated in protected areas or in private territories. Our study did not involve endangered or protected species.

### Screening for parasites

In mid-June, each specimen was examined under a stereomicroscope to detect whether it was infested with parasites. First, a ladybird was examined externally from above and from below to determine the presence of ectoparasites. Then, its elytra were removed to detect whether the tergites were infested with mites or other parasites. Then, the abdomen of each specimen was dissected to find endoparasites. This method allows the detection of different parasites in one specimen. The dissection was performed in 0.9% NaCl solution. The collected nematodes were heat-killed (at 65°C) and fixed with TAF solution. Permanent slides of the nematodes in anhydrous glycerin were prepared following the Seinhorst method [27]. Sixty permanent slides were made. These slides and TAF-fixed nematodes are kept in the Helminthological Museum of the Russian Academy of Sciences (Moscow). Morphometric analysis of common nematode body features was carried out on five fixed nematode specimens of each life stage using a Zeiss Jenaval microscope. Photographs were taken using a Leica 5500B microscope. Statistical analyses were conducted using the Systat 10.2 program.

### Identification

The identification of the parasites was primarily based on morphological features. The identification of *P*. *bifurcatus* was confirmed using nucleotide sequence analysis. The nematodes recovered from the ladybird hemocoel were individually frozen in sterile 0.7 ml Eppendorf tubes for DNA extraction, which was performed according to Holterman et al. [28]. The worm-lysis solution (950 μl of a mixture of 2 ml of 1 M NaCl, 2 ml of 1 M Tris-HCl (pH 8), 5.5 ml of deionized water, 10 μl of mercaptoethanol and 40 μl of proteinase K, 20 mg ml^–1^) was prepared immediately before DNA extraction. Aliquots of 25 μl of sterile water and 25 μl of the worm-lysis solution were added to each tube with a nematode and incubated at 65°C for 90 min. The tubes containing the homogenate were then incubated at 99°C for 5 min to deactivate the proteinase K. Approximately 1.0 μl of the homogenate was used as the PCR template.

PCR reactions were performed using the Encyclo Plus PCR kit (Evrogen, Moscow, Russia) according to the manufacturer’s protocol. The primer pairs Nem18S_F (5’-CGC GAA TRG CTC ATT A CA ACA GC-3’) and 26R (5’-CAT TCT TGG CAA ATG CTT TCG-3’) were used to obtain a partial (approximately 900 bp long) sequence of the 5’ half of the mitochondrial 18S rDNA [29]. The PCR cycling parameters included primary denaturation at 94°C for 5 min followed by 34 cycles at 94°C for 45 s, 54°C for 60 s and 72°C for 1 min, followed by postamplification extension at 72°C for 3 min.

A pair of primers, D2A (5’-ACA AGT ACC GTG AGG GAA AGT TG -3’) and D3B (5’-TCG GAA GGA ACC AGC TAC TA-3’), was used to amplify an approximately 800 bp long sequence of the D2D3 expansion segment of 28S rDNA [30]. The PCR cycling parameters included denaturation at 95°C for 3 min, followed by 35 cycles at 94°C for 30 s, 54°C for 35 s, and 72°C for 70 s, followed by postamplification extension at 72°C for 5 min.

The PCR products were visualized in 1% agarose gel. Then, the bands containing PCR products obtained were excised from 0.8% agarose gel for the DNA extraction using the Wizard SV Gel and PCR Clean-Up System (Promega, Madison, USA). The samples were directly sequenced using the same primers as those used for the primary PCR. The sequencing with each primer was made twice. The sequences obtained from the reading with the opposite primers were aligned manually after checking the sequence reads in Chromas 2.4.4 (free software from Technelysium Pty Ltd). Similar sequences were identified in NCBI GenBank using the BLAST algorithm [31] and were found belonging to the superfamily Sphaerularioidea, and all the available data for this taxon were selected for the further phylogenetic analysis. Clustal X [32] was used to align the obtained sequences, and then GeneDoc 2.7. [33] was used to remove the flanking parts of the sequences with different lengths to obtain a rectangular data matrix (726 characters in 18S matrix and 806 in 28S matrix). For the phylogenetic analyses, the program MEGA 7.0.14 [34] was used. Neighbor joining (NJ) analysis was conducted under Maximum Composite Likelihood method, with substitutions to include both transitions and transversions and uniform rates among sites. Maximum parsimony (MP) analysis was conducted with Subtree-Pruning-Regrafting as the tree topology search. Rapid bootstrapping was conducted with 1000 replicates for the MP and NJ analyses. The jModelTest2 [35] was used to select the best-fit model of nucleotide substitution. The General Time Reversible model (gamma distributed with invariant sites—GTR+G+I) has been found to be the best for both for 18S and 28S rDNA data. Maximum Likelihood (ML) analysis has also been performed with MEGA 7.0.14 with Nearest-Neighbor-Interchange as tree inference option and with rapid bootstrapping under 500 replicates. The obtained sequences have been deposited in GenBank as MH718837 for the 18S rDNA sequence and MH722215 for the 28S rDNA.

### Collection and external examination of other ladybird species

Since fungi of the genus *Hesperomyces* can parasitize not only *Ha*. *axyridis* but many other ladybirds [36, 37], we decided to examine other ladybirds collected using the same methods and in the same localities as *Ha*. *axyridis*. Four hundred specimens of ladybirds of 29 other species (both native and introduced) were collected and screened for ectoparasitic fungi (Table 1).

**Table 1:** The list of other ladybird species screened for ectoparasitic fungi.

Species	Veseloe (43.41, 39.98),	Central District (43.58, 39.73),	Adler (43.42, 39.94),	Valley of Agur river (43.56, 39.83),	Golovinka (43.79, 39.47),	Razbityj Kotel (43.69, 39.73)
*Adalia bipunctata* (Linnaeus)	*1*	*3*	*2*	*1*	*1*	
*Anisosticta novemdecimpunctata* (Linnaeus)		*1*				
*Calvia decemguttata* (Linnaeus)		*1*				
*Chilocorus bipustulatus* (Linnaeus)		*2*				
*Chilocorus renipustulatus* (Scriba)		*6*				
*Coccinella quinquepunctata* Linnaeus		*2*		*1*		
*Coccinella septempunctata* Linnaeus	*5*	*10*	*6*	*5*	*2*	*1*
*Coccinula quatuordecimpustulata* (Linnaeus)	*15*	*10*	*12*	*12*	*3*	*8*
*Cryptolaemus montrouzieri* Mulsant	*20*	*15*				
*Exochomus quadripustulatus* (Linnaeus)		*3*				
*Halyzia sedecimguttata* (Linnaeus)	*1*	*2*	*1*			
*Harmonia quadripunctata* (Pontoppidan)	*5*	*6*	*4*	*3*		
*Hippodamia variegata* (Goeze)	*6*	*15*	*8*	*7*		
*Lindorus lophanthae* (Blaisdell)		*2*				
*Nephus bipunctatus* (Kugelann)	*3*	*5*	*8*	*1*	*1*	*2*
*Parexochomus nigromaculatus* (Goeze)		*3*				
*Propylea quatuordecimpunctata* (Linnaeus)	*5*	*20*	*6*	*10*	*4*	*3*
*Psyllobora vigintiduopunctata* (Linnaeus)	*1*	*2*		*1*	*1*	
*Rodolia cardinalis* (Mulsant)	*2*					
*Scymnus frontalis* (Fabricius)	*4*	*10*	*3*		*2*	*1*
*Scymnus haemorrhoidalis* Herbst	*5*	*12*	*8*	*5*	*5*	
*Scymnus interruptus* (Goeze)		*5*				
*Scymnus subvillosus* (Goeze)	*3*	*2*	*3*			
*Scymnus suturalis* Thunberg		*4*	*1*			
*Serangium montazerii* Fürsch	*5*	*2*				
*Stethorus pusillus* (Herbst)		*2*				
*Subcoccinella vigintiquatuorpunctata* (Linnaeus)	*19*	*7*				
*Tytthaspis sedecimpunctata* (Linnaeus)	*1*	*1*			*1*	
*Vibidia duodecimguttata* (Poda)	*1*		*1*			

## Results

We found three parasitic species on the examined *Ha*. *axyridis* adults: *He*. *virescens* (Ascomycota: Laboulbeniales, Laboulbeniaceae), *P*. *bifurcatus* (Nematoda: Tylenchida Allantonematidae) and an unknown species belonging to Mermithida (Nematoda). All examined *Ha*. *axyridis* specimens were free of the parasitic mite *Coccipolipus hippodamiae*.

### Hesperomyces virescens

**Fig 2:**
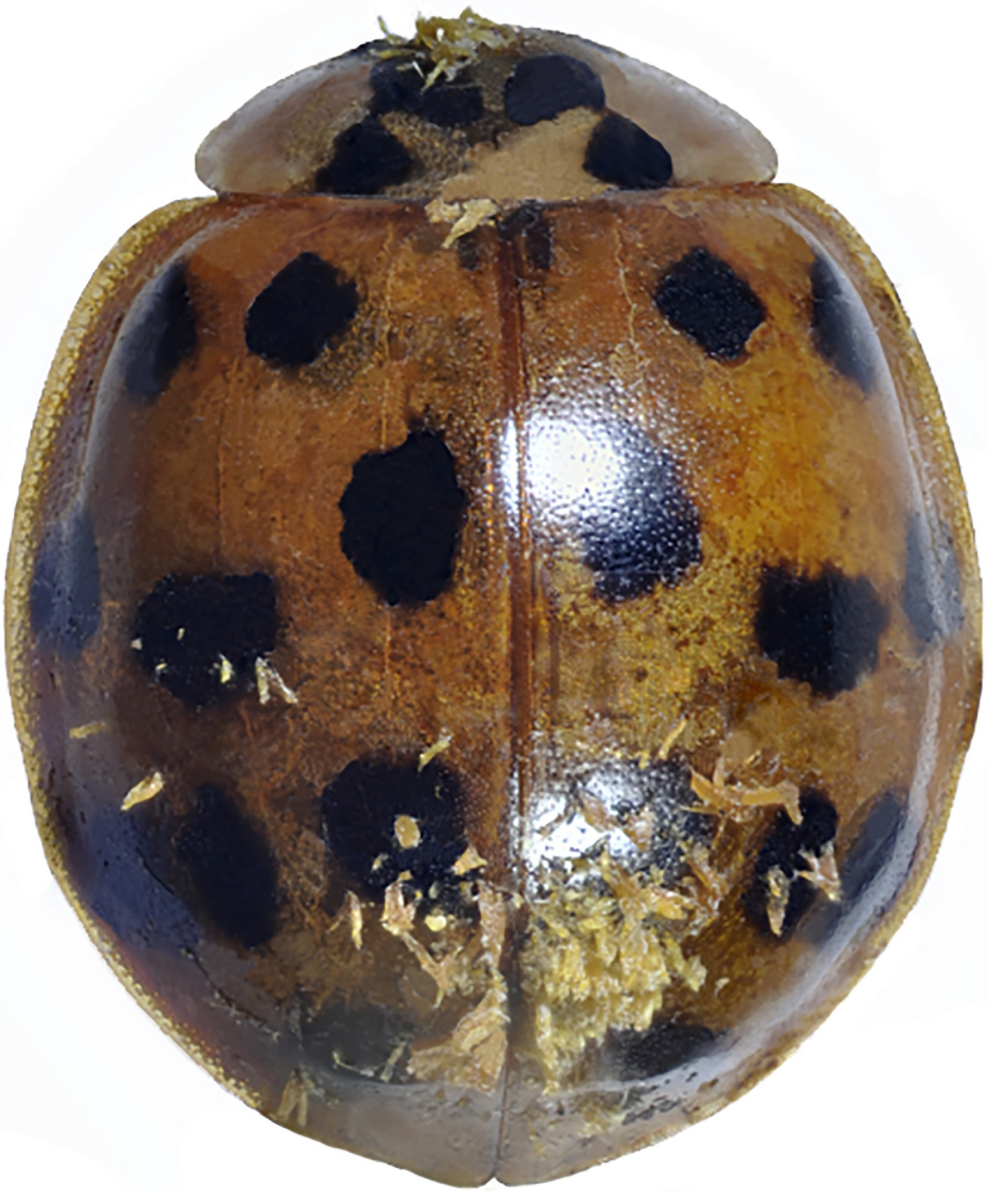
*Hesperomyces virescens* on *Ha*. *axyridis*:) ladybird covered with thalli.

The characteristic yellowish-greenish thalli of *He*. *virescens* were detected on the host integuments (Fig 2). Their morphology corresponds to the description by De Kesel [36]. Identification was confirmed by mycologist E. Yu. Blagoveshchenskaya from Moscow State University.

Specimens of *Ha*. *axyridis* infested with *He*. *virescens* were found at all six localities from which the beetles were collected. The distance between the most western locality (Golovinka) and the most eastern (Veseloe) is more than 60 km. Thalli were found on 112 (53%) adults of *Ha*. *axyridis* (Table 2) and were located on the elytra, pronotum, sternites, legs and mouthparts of the beetles. Interestingly, even specimens covered with a large number of fungal thalli moved actively. Males were infested more often than females (62% and 49%, respectively), but Fisher’s exact test and Pearson’s chi-square test showed that these differences are not statistically significant (S2 Appendix). Our sampling is just a temporal snapshot of parasite prevalence. Seasonal and between-year variation in abundances might alter the relative proportions of parasites and cause sex differences in the infection of *Ha*. *axyridis* [1]. No signs of ectoparasitic fungi were detected on the 400 examined adults of other ladybird species.

**Table 2:** Infestation of *Harmonia axyridis* with *Hesperomyces virescens* and *Parasitylenchus bifurcatus*.

	Number of specimens without parasites	Number of specimens infested with only *He*. *virescens*	Number of specimens infested with only *P*. *bifurcatus*	Number of specimens infested with *P*. *bifurcatus* & *He*. *virescens*	Total
Males	22	37	1	1	61
Females	69	67	9	7	152
Total	91	104	10	8	213

### Parasitylenchus bifurcatus

Parasitic nematodes of the family Allantonematidae were found in 18 specimens of *Ha*. *axyridis* collected at three out of the six sampling locations (see Fig 1). Males, vermiform (infective) females and subsequent generation parasitic females were found in the sampled beetles. The number of females of the subsequent generations varied from 5 to 32 per beetle. The number of vermiform nematode specimens varied considerably, sometimes being as high as approximately two hundred per beetle.

**Fig 3:**
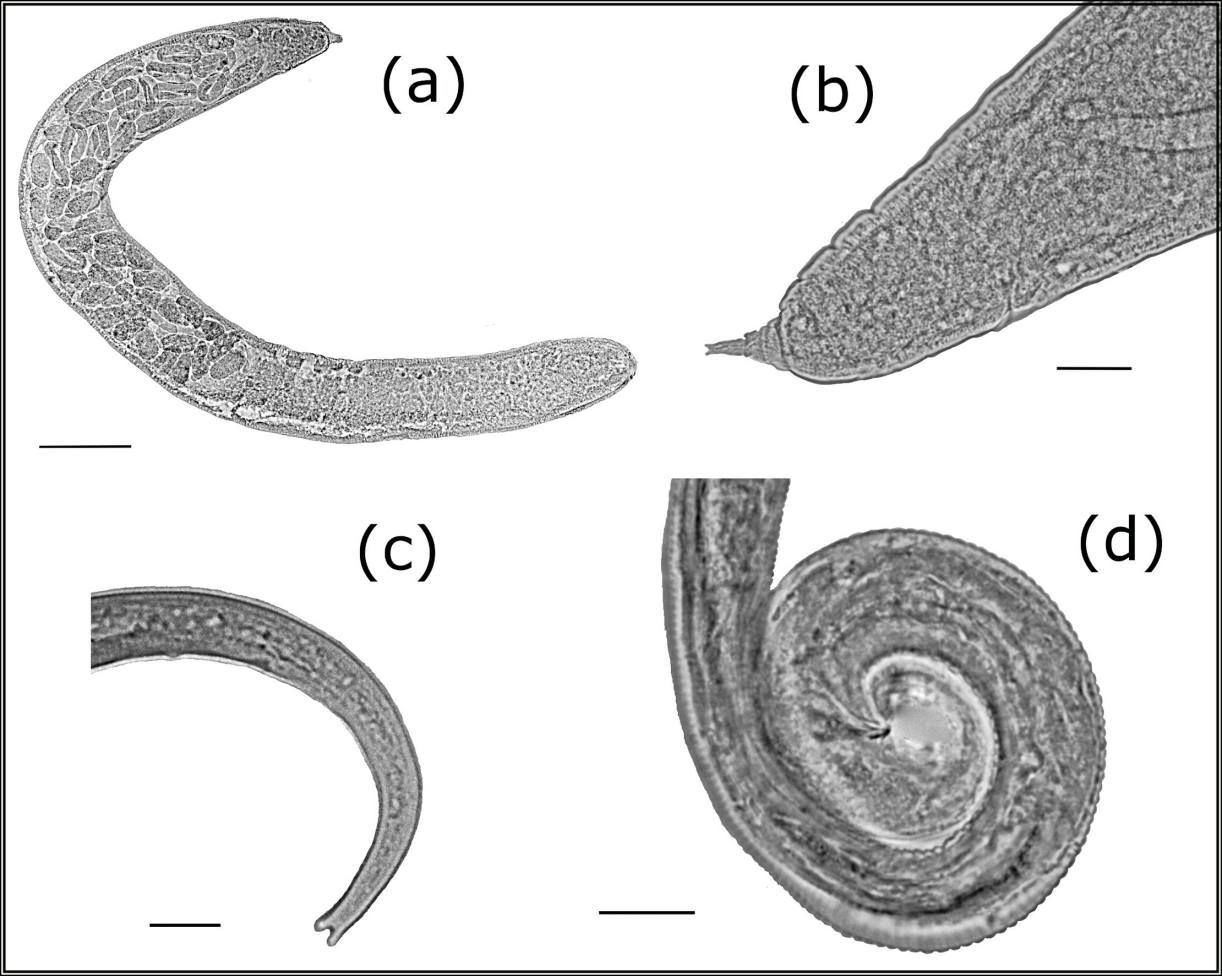
*Parasitylenchus bifurcatus*: (a) subsequent generation parasitic female of *P*. *bifurcatus* (bar 98 μm), (b) subsequent generation parasitic female, tail (bar 28 μm), (c) vermiform (infective) female, tail (bar 12 μm), (d) male, tail (bar 12 μm).

The nematodes were identified as *P*. *bifurcatus* (Table 3). The characteristic features of *P*. *bifurcatus* nematodes are as follows: a straight stylet lacking basal thickenings, a forked tail tip in the vermiform females and juvenile males, spicules straight, wedge-shaped or triangular, with narrow bursa and gubernaculum (Fig 3). We have found that the subsequent generation of parasitic females also has forked tail tip.

**Table 3:** Morphometric characteristics of specimens of the nematode *P*. *bifurcatus* isolated from the ladybird *Ha*. *axyridis* collected in Sochi. Raw morphometric data can be found in the supplementary material (S3 Appendix).

Character	Parasitic females of the subsequent generation (n = 5)	Vermiform (infective) females (n = 5)	Males (n = 5)
Body length, μm	1118.0 (930.0–1660.0)	590.0 (530.0–670.0)	403.0 (396.0–480.0)
Body width, μm	124.0 (85.0–184.0)	12.6 (12.0–13.0)	15.0(14.0–16.0)
Stylet length, μm		11.5 (11.0–12.0)	9.0 (8.0–11.0)
Distance from head to excretory pore, μm	155.4 (141.0–165.0)	50.6 (42.0–57.0)	66.0(62.0–75.0)
Vulva position, %	89.4 (88.0–93.0)	88.0 (87.0–90.0)	
Tail length, μm	39 (25.0–48.0)	33.2 (30.0–37.0)	34.4(25.5–40.0)
Spicule length, μm			12(11–13)

**Fig 4:**
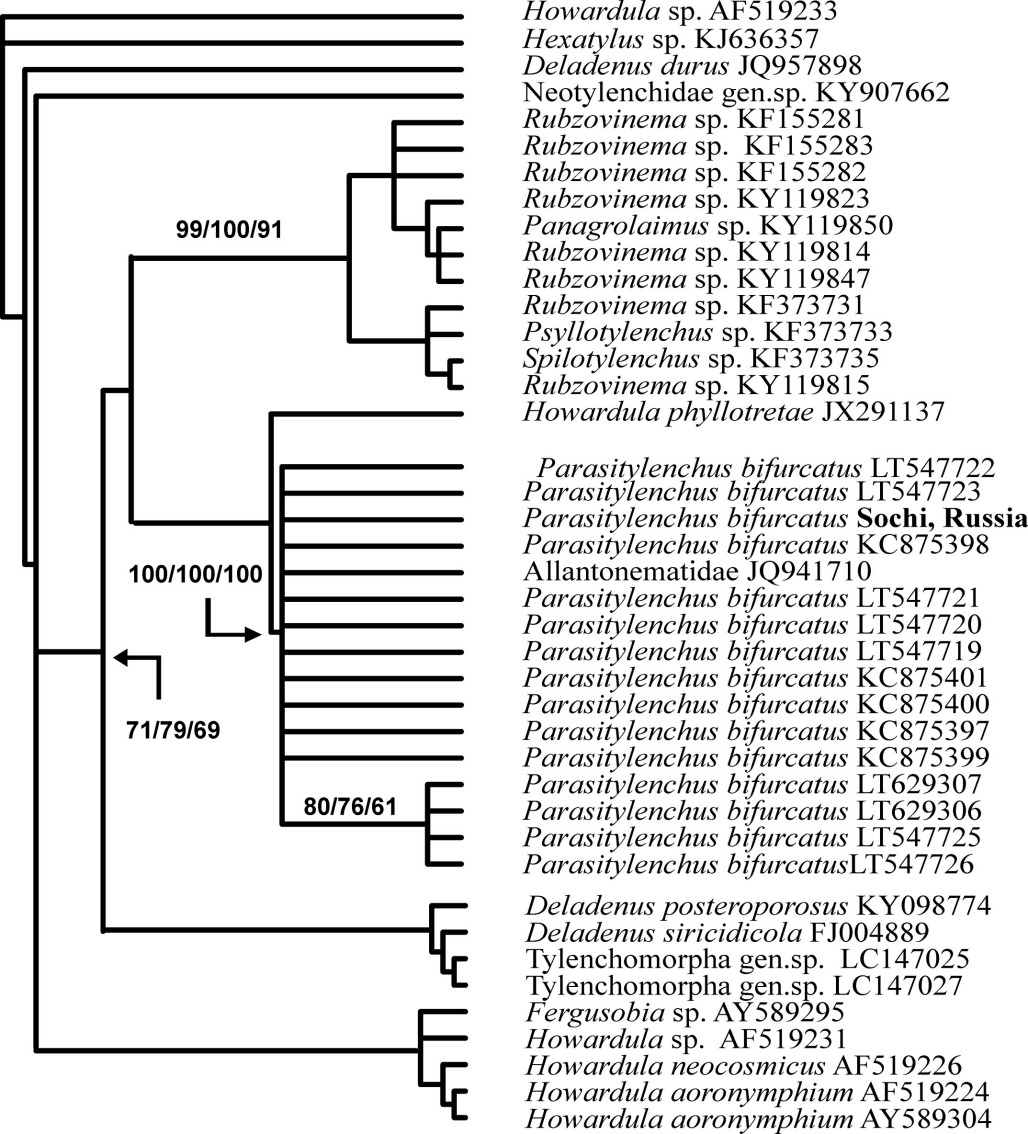
The relationships of *Parasitylenchus bifurcatus* from Sochi, Russia, with other groups of insect-associated tylenchids inferred from the analysis of partial 18S rDNA. Bootstrap support is given near the corresponding nodes in the format MP/NJ/ML.

A BLAST search for similar nucleotide sequences in NCBI GenBank was performed for all three obtained sequences. These were the sequences of different clones and isolates of *P*. *bifurcatus* that were found to be the closest to the obtained 18S rDNA sequence of nematodes from *Ha*. *axyridis* ladybirds from the Russian Caucasus. All the similar sequences detected by BLAST search were downloaded and used for comparison. Using all methods of analysis, the obtained sequence was a member of a strongly supported clade (100% bootstrap support) consisting of *P*. *bifurcatus* sequences plus a sequence from an unidentified Allantonematidae (Fig 4). The obtained sequence of the *Ha*. *axyridis* parasite from the Russian Caucasus was 100% identical to some published sequences of *P*. *bifurcatus* (e.g., clones ‘3l4j’, ‘3j4i’ and ‘PaTyBif1’). Remarkably, the sequence of an unidentified allantonematid nematode found in 2010 in Germany in the hemocoel of *Ha*. *axyridis* (E.L. Rhule, unpublished), which was deposited as JQ941710, was found to be identical to the sequence obtained in our study. It seems that the nematodes from Germany also belong to the species *P*. *bifurcatus*. All other known 18S rDNA sequences of *P*. *bifurcatus* differ from our sequence in one or two nucleotides. Several clades demonstrated close but not securely resolved relationships with the clade containing the sequences of *P*. *bifurcatus*. All methods of analysis show that the 18S rDNA sequence of *Howardula phyllotretae* Oldham, 1933 is in sister relationships with those of the *P*. *bifurcatus* clade (Fig 4). One such clade consists of the sequences of parasitic tylenchids of fleas: *Rubzovinema* Slobodyanyuk, 1991, *Spilotylenchus* Launay, Deunff & Bain, 1983 and *Psyllotylenchus* Poinar & Nelson, 1973 (Fig 4). Another related clade is represented by species of *Deladenus* Thorne, 1941 and an unidentified Tylenchomorpha gen. sp. Other sequences of *Howardula* Cobb, 1921 were most distant from *P*. *bifurcatus* (differing in 59–60 bp) in our analysis and served as a root for the obtained cladograms.

**Fig 5:**
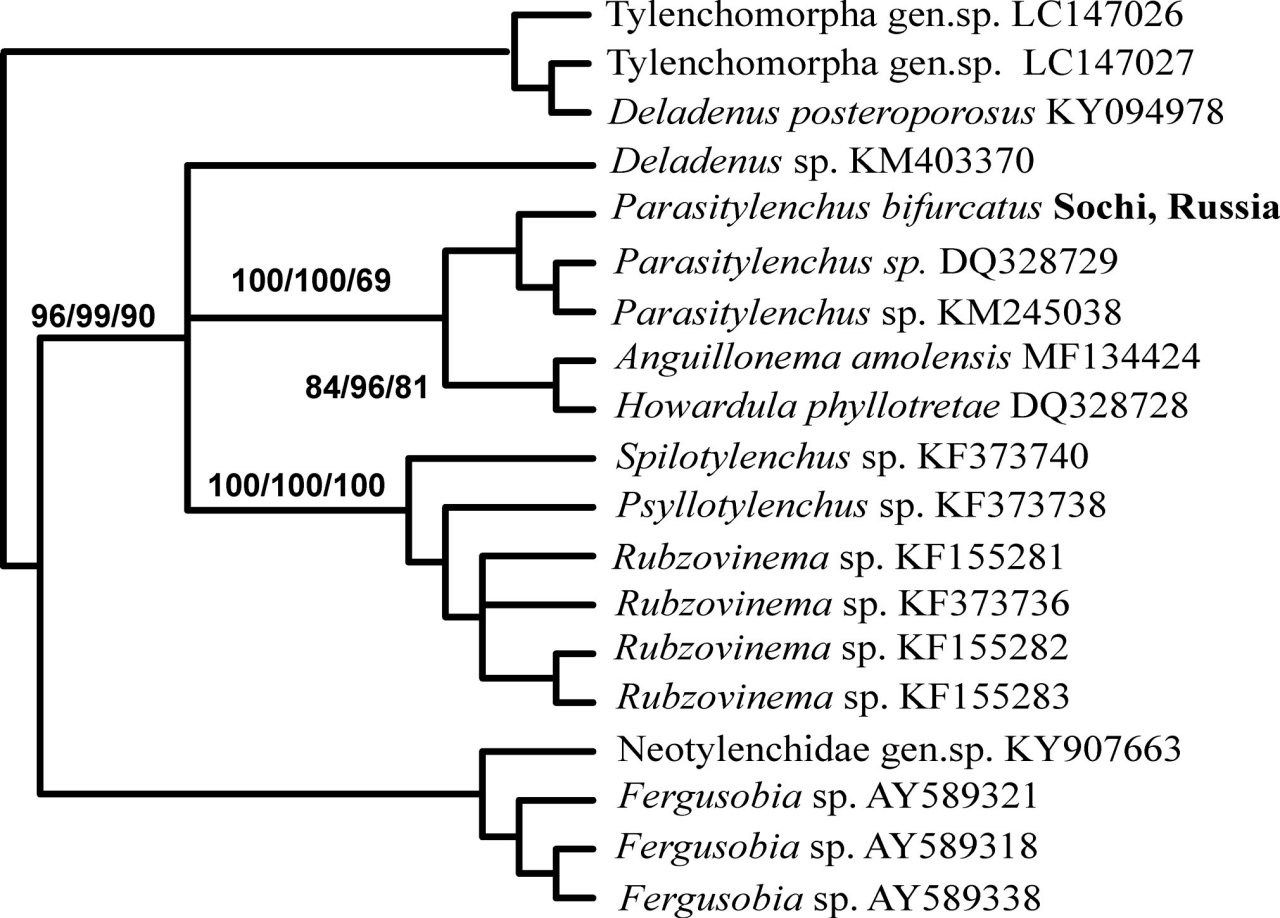
The relationships of *Parasitylenchus bifurcatus* from Sochi, Russia, with other groups of insect-associated tylenchids inferred from the analysis of partial 28S rDNA. Bootstrap support is given near corresponding nodes in the format MP/NJ/ML.

The 28S rDNA sequences of entomoparasitic tylenchids known and deposited in NCBI GenBank are less numerous than those from 18S rDNA data. The obtained cladogram demonstrates the close relationships between the sequence of the studied nematode and two other sequences obtained for unidentified species of *Parasitylenchus*: DQ328729 and KM245038 (Fig 5). These sequences are related to parasites of bark beetles in Russia and the Czech Republic, respectively. At the level of nucleotide differences, these two *Parasitylenchus* sequences are closest to those of nematodes from *Ha*. *axyridis* (87 and 90 bp), while the differences in nucleotides from those in the sequences of all other studied entomoparasitic tylenchids exceed 100 bp. The sequence of *Howardula phyllotretae* together with that of *Anguillonema amolensis* Mobasseri, Pedram et Pourjam, 2017 form a clade that is in a sister position to that of the *Parasitylenchus* Micoletzky, 1922 clade based on all methods of analysis (Fig 5). As in the 18S rDNA cladogram, the sequences of flea parasites (*Rubzovinema*, *Spiolotylenchus*, *Psyllotylenchus*) form a well-supported clade (Fig 5). The relationships of this latter clade with the *Parasitylenchus* (*Howardula phyllotretae* + *Anguillonema amolensis*) clade are strongly supported.

**Fig 6:**
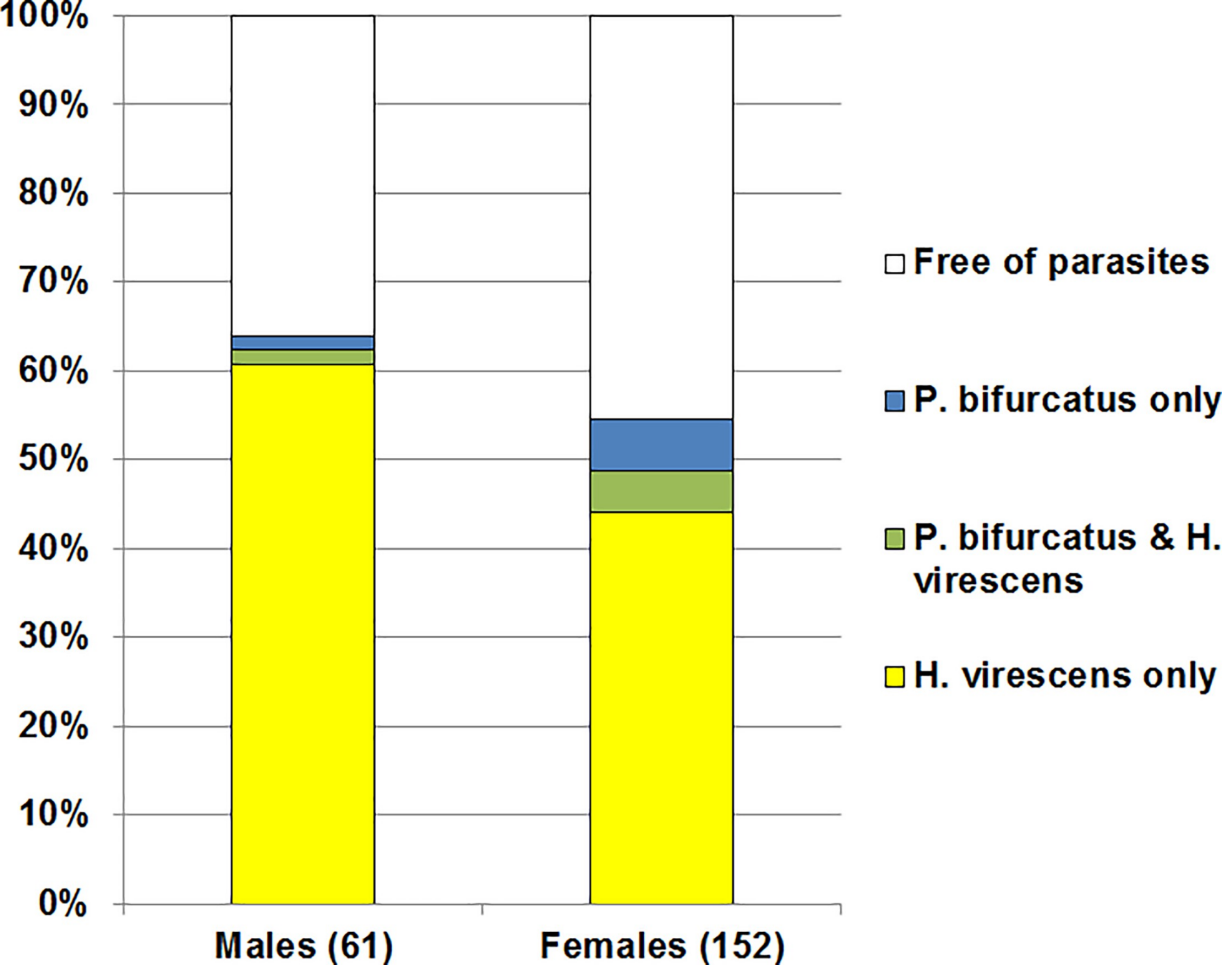
Proportion of *Harmonia axyridis* adults infested with *Hesperomyces virescens* and *Parasitylenchus bifurcatus*. The number of examined specimens is indicated in brackets.

*Parasitylenchus bifurcatus* nematodes were found in 8% of the dissected adults of *Ha*. *axyridis*. The prevalence in females and males was 10% and 3%, respectively, but Fisher’s exact test and Pearson’s chi-square test showed that these differences are not statistically significant. *Parasitylenchus bifurcatus* was also found in specimens infested with *He*. *virescens* and free of fungi, and no correlation in the infestation of specimens with *P*. *bifurcatus* and *He*. *virescens* was observed (Fig 6).

One specimen of a nematode belonging to the order Mermithida was found in the only ladybird specimen sampled in Veseloe.

## Discussion

### Sequence analysis of *P*. *bifurcatus*

The obtained nucleotide sequences are important for the confirmation of the primary parasitic nematode identification based on morphological features. Several sequences of the 18S rDNA of *P*. *bifurcatus* are deposited in NCBI GenBank, and newly obtained sequences for the specimens from Sochi are identical with some of these. Unlike the 18S rDNA data, those for the large ribosomal subunit (28S rDNA) of *Parasitylenchus* nematodes are quite scarce and can only suggest the sufficient informative value of this locus for phylogenetic studies of entomoparasitic tylenchids. These data effectively demonstrated the clustering of the *P*. *bifurcatus* sequence with two known sequences from *Parasitylenchus* nematodes. The results of both the 18S and 28S analyses revealed some incongruence in the contemporary taxonomy of these nematodes. It is obvious from the obtained phylogenetic reconstructions that some genera of parasitic nematodes are paraphyletic: we can find the species of *Howardula* in three clades of the 18S rDNA cladogram and *Deladenus* sequences in two clades of the 18S and 28S rDNA cladograms.

### Both *He*. *virescens* and *P*. *bifurcatus* are alien to the Caucasus

We believe that the two parasitic species (*He*. *virescens* and *P*. *bifurcatus*) are not native to the Caucasus. First, *He*. *virescens* and *P*. *bifurcatus* were absent from the region and were first found soon after the first record of their host. The location of the current records of the parasites (Sochi) is situated more than 1000 km from the nearest known localities of both parasitic species (Bulgaria for *He*. *virescens* [19] and Poland for *P*. *bifurcatus* [1]). Second, the analysis of the phylogenetic relationships of *P*. *bifurcatus* based on 18S rDNA demonstrated the complete identity of the 18S rDNA sequence of these nematodes from the Russian Caucasus with some strains or clones found in Western and Southern Europe. Cautiously, such identity can be considered to be an indication of the possible transfer of parasites together with their insect hosts from the Western part of Eurasia. Third, although other species of Coccinellidae were not dissected to find nematodes, *Ha*. *axyridis* is the only known host of *P*. *bifurcatus*, and there is no reason to suggest that a *Ha*. *axyridis* individual was infected from other ladybird species. The examination of 400 specimens of 29 other ladybird species collected from the same localities as *Ha*. *axyridis* (Table 1) showed that they were not infested with *He*. *virescens*.

*Hesperomyces virescens* is found to be widespread and common on *Ha*. *axyridis* on the Black Sea coast of the Caucasus. However, despite the high prevalence of *He*. *virescens* on *Ha*. *axyridis*, we did not find it on other potential hosts in the Caucasus. No signs of Laboulbeniales ectoparasites have been detected on other ladybird species, although seven of them were indicated by Ceryngier and Twardowska [38] as hosts of *He*. *virescens* in other regions: *Adalia bipunctata*, *Chilocorus bipustulatus*, *Chilocorus renipustulatus*, *Coccinula quatuordecimpustulata*, *Propylea quatuordecimpunctata*, *Psyllobora vigintiduopunctata* and *Tytthaspis sedecimpunctata*. The same situation was previously observed by A. De Kesel in Europe despite some potential hosts overwintering at the same sites with *Ha*. *axyridis* [1].

### The sources of the invasion of *Ha*. *axyridis* in the Caucasus

Since *Ha*. *axyridis* was released for the biological control of pests in the Caucasus, it is unclear whether the population of *Ha*. *axyridis* originated as a result of these releases or as a result of the expansion of its European range [12, 17]. The study of parasites has shed light on this question. Both *He*. *virescens* and *P*. *bifurcatus* only affect adults, do not occur on other life stages and exclusively spread through the activities of the host [23]. Transmission takes place only from adult to adult; therefore, direct contact between different generations of beetles is necessary to maintain the life cycle of the parasites. Since different generations are kept separately in the laboratory culture [39], it is free of parasites. Therefore, the detection of *He*. *virescens* and *P*. *bifurcatus* indicates that the population of *Ha*. *axyridis* in the Caucasus did not derive exclusively from specimens released from laboratory culture. At least some of the ancestors of the Caucasian population of *Ha*. *axyridis* are from the European invasive range. On the other hand, the admixture of released specimens is not excluded, since *Ha*. *axyridis* was released in Sochi for several decades. Such complex invasion scenaria are commonplace for alien insects in general [40] and for *Ha*. *axyridis* in particular [41]. This case and some other recent studies [2] confirm that parasitological analysis is a promising approach for revealing invasion routes.

Roy et al. [20] proposed that connection between *He*. *virescens* and *Ha*. *axyridis* coupled with the rapid expansion of *Ha*. *axyridis* globally suggests that this parasite will continue to spread throughout the rest of the world. The spread of *He*. *virescens* to the Caucasus confirms this suggestion.

It was shown by Haelewaters et al. [1] that there was a time lag between the invasion of *H*. *axyridis* and that of its parasites in the Netherlands and North America. The same phenomenon was recorded for other *Herpomyces* ectoparasitic fungi on other invasive hosts [42]. The population of *H*. *axyridis* in the Caucasus was detected in 2012 and first screened for parasites in 2018. It is unknown whether the ladybirds were infested with the parasites before 2018. There are no specimens of *Ha*. *axyridis* collected in the Caucasus before 2018 in Zoological Institute of Russian Academy of Sciences (St,-Petersburg) and Zoological Museum of Moscow State University (Moscow). Therefore, it is impossible to determine whether the first founders of the Caucasian population were infested with the parasites or whether the parasites were introduced by specimens of *Harmonia axyridis* that arrived later from Europe.

### Coinfection of *Ha*. *axyridis* with *He*. *virescens* and *P*. *bifurcatus*

The coinfections of *H*. *axyridis* by different parasites is poorly studied. Riddick [43] observed *Ha*. *axyridis* parasitized with *He*. *virescens* and *Coccipolipus hippodamiae*. Coinfection of *Ha*. *axyridis* with *He*. *virescens* and *P*. *bifurcatus* was recorded in the Netherlands, and a positive association between these parasites was detected that correlated with a reduced number of live beetles [25]. No correlation between infestation by *He*. *virescens* and *P*. *bifurcatus* has yet been recorded in the Caucasus. However, the number of collected specimens was small, so the possibility of such a correlation cannot be ruled out. The study of coinfection of *Ha*. *axyridis* by these two parasites is an intriguing subject for future studies, since coinfections might result in lower survival rates.

### Infestation of *Ha*. *axyridis* with a mermithid nematode

We have made the first record of infestation of *Ha*. *axyridis* with a representative of the order Mermithida. Infestations of coccinellids with mermithid nematodes are quite rare [44]. Linstow [45] reported *Mermis nigrescens* Dujardin (Mermithida: Mermithidae) to be a parasite of *Coccinella septempunctata*. The infestation of *Adonia variegata*, *C*. *septempunctata* and *Semiadalia undecimnotata* with unidentified mermithids in southeast France was reported by Iperti [46]. Kaiser and Nickle [47] described the infestation of *Coccinella septempunctata* with *Hexamermis* sp. in Styria, Austria. A study focused on the search for parasitic nematodes of this order in the Sochi area and their identification is planned for the future.

## Conclusions

1. The population of *Ha*. *axyridis* in the Caucasus, which was recently invaded by this species, is infested with two parasite species that were recorded in the Caucasus and Russia for the first time: *He*. *virescens* and *P*. *bifurcatus*. It is likely that these parasites appeared in the region as a result of coinvasion with *Ha*. *axyridis*.
2. The population of *Ha*. *axyridis* in the Caucasus appeared as a result of the expansion of the European range, as the 18S rDNA sequences of Caucasian *P*. *bifurcatus* and those from Western Europe are 100% identical. It could not have derived exclusively from specimens released for the biological control of pests because laboratory cultures are free of these parasites.
3. Although *Hesperomyces virescens* develops on many ladybird species in other regions, its only known host in the Caucasus is *Ha*. *axyridis*.
4. An unidentified species of the order Mermithida was recorded on *Ha*. *axyridis* in the Caucasus. This is the first documented case of the infestation of *Ha*. *axyridis* by a parasitic nematode of this order in nature.

## Supporting information

S1 Appendix Localities of *Harmonia axyridis* and its parasites *Hesperomyces virescens* and *Parasitylenchus bifurcatus* in the Caucasus. (XLS)

S2 Appendix Statistical analysis of data on the infestation of males and females of *Harmonia axyridis* with *Hesperomyces virescens* and *Parasitylenchus bifurcatus* in the Caucasus. (DOC)

S3 Appendix Raw morphometric data on *Parasitylenchus bifurcatus*. (DOCX)
