# Supplementary material for: Coinvasion by the ladybird Harmonia axyridis (Coleoptera: Coccinellidae) and its parasites, Hesperomyces virescens (Ascomycota: Laboulbeniales) and Parasitylenchus bifurcatus (Nematoda: Tylenchida, Allantonematidae), in the Caucasus
Source: PLoS One. 2018 Nov 29;13(11):e0202841. doi: 10.1371/journal.pone.0202841 (PMC6264875; doi:10.1371/journal.pone.0202841)
Supplement: S2 Appendix — (DOC) [file pone.0202841.s002.doc]

**Supporting information S2**

| **Name of the article: Coinvasion by the ladybird Harmonia axyridis and its parasites, Hesperomyces virescens (fungus) and Parasitylenchus bifurcatus (nematode), in the Caucasus** |
| --- |
| **Journal: PLoS One** |
| **Authors: Orlova-Bienkowskaja1 MJ, Spiridonov SE, Butorina NN, Bieńkowski AO** |

Statistical treatment of data on infestation of males and females

**Infestation of *H. axyridis* with *Hesperomyces virescens***

Rows: 0 –males, 1 – females

Columns: 0 – not infested, 1 - infested

Row percents

SEX (rows) by HV (columns)

0 1 Total N

+-----------------+

0 | 37.705 62.295 | 100.000 61

1 | 51.316 48.684 | 100.000 152

+-----------------+

Total 47.418 52.582 100.000

N 101 112 213

Test statistic Value df Prob

Pearson Chi-square 3.234 1.000 0.072

Fisher exact test (two-tail) 0.095

**Infestation of *H. axyridis* with *Parasitylenchus bifurcatus***

Rows: 0 –males, 1 – females

Columns: 0 – not infested, 1 - infested

SEX (rows) by PB (columns)

0 1 Total N

+-----------------+

0 | 96.721 3.279 | 100.000 61

1 | 89.474 10.526 | 100.000 152

+-----------------+

Total 91.549 8.451 100.000

N 195 18 213

Test statistic Value df Prob

Pearson Chi-square 2.956 1.000 0.086

Fisher exact test (two-tail) 0.105
