# Supplementary material for: Coinvasion by the ladybird Harmonia axyridis (Coleoptera: Coccinellidae) and its parasites, Hesperomyces virescens (Ascomycota: Laboulbeniales) and Parasitylenchus bifurcatus (Nematoda: Tylenchida, Allantonematidae), in the Caucasus
Source: PLoS One. 2018 Nov 29;13(11):e0202841. doi: 10.1371/journal.pone.0202841 (PMC6264875; doi:10.1371/journal.pone.0202841)
Supplement: S3 Appendix — (DOCX) [file pone.0202841.s003.docx]

**Parasitic females of the subsequent generation (n= 5)**

| Character | Female | | | | | Mean (range) |
| --- | --- | --- | --- | --- | --- | --- |
|  | №1 | №2 | №3 | №4 | №5 |  |
| Body length, µm | 1005.0 | 990.0 | 930.0 | 1005.0 | 1660.0 | **1118.0 (930.0-1660.0)** |
| Body width, µm | 122.0 | 105.0 | 85.0 | 124.0 | 184.0 | **124.0 (85.0-184.0)** |
| a | 8.2 | 9.4 | 10.9 | 8.1 | 9.0 | **9.1(8.1-10.9)** |
| Head to excretory pore distance, µm | 158.0 | 152.5 | 141.0 | 160.5 | 165.0 | **155.4 (141.0-165.0)** |
| Head to vulva distance, µm | 885.0 | 882.0 | 828.0 | 885.0 | 1545.0 | **1005.0(828.0-1545.0)** |
| Vulva position, % | 88 | 89 | 89 | 88 | 93 | **89.4 (88.0-93.0)** |
| Tail length, µm | 45,0 | 25.0 | 30.0 | 47.0 | 48.0 | **39.0 (25.0-48.0)** |

**Vermiform (infective) females (n= 5)**

| Character | Female | | | | | Mean (range) |
| --- | --- | --- | --- | --- | --- | --- |
|  | №1 | №2 | №3 | №4 | №5 |  |
| Body length, µm | 530.0 | 535.0 | 670.0 | 610.0 | 605.0 | **590.0 (530.0-670.0)** |
| Body width, µm | 12.0 | 12.0 | 13.0 | 13.0 | 13.0 | **12.6 (12.0-13.0)** |
| a | 44.2 | 44.6 | 51.5 | 46.9 | 46.5 | 46.7(44.2-51.5) |
| Stylet length , µm | 11.0 | 11.0 | 12.0 | 11.5 | 12.0 | **11.5 (11.0-12.0)** |
| Head to excretory pore distance, µm | 42.0 | 45.0 | 57.0 | 55.0 | 54.0 | **50.6 (42.0-57.0)** |
| Head to vulva distance, µm | 463.0 | 465.0 | 603.0 | 545.0 | 527.0 | **520.6(463.0-603.0)** |
| Vulva position, % | 87 | 87 | 90 | 89 | 87 | **88.0 (87.0-90.0)** |
| Tail length, µm | 30.0 | 34.0 | 37.0 | 32.5 | 32.5 | **33.2 (30.0-37.0)** |

**Males (n= 5)**

| Character | Male | | | | | Mean (range) |
| --- | --- | --- | --- | --- | --- | --- |
|  | №1 | №2 | №3 | №4 | №5 |  |
| Body length, µm | 396.0 | 480.0 | 397.0 | 396.0 | 396.0 | **413.0 (396.0-480.0)** |
| Body width, µm | 14.0 | 16.0 | 15.0 | 16.0 | 14.0 | **15.0(14.0-16.0)** |
| a | 28.3 | 30.0 | 26.5 | 24.7 | 28.3 | **27.6(24.7-30.00)** |
| Stylet length , µm | 8.0 | 11.0 | 11.0 | 8.0 | 9.0 | **9.0 (8.0-11.0)** |
| Head to excretory pore distance, µm | 65.0 | 75.0 | 64.0 | 62.0 | 64.0 | **66.0(62.0-75.0)** |
| Spicule length, µm | 11.0 | 13.0 | 11.0 | 12.0 | 13.0 | **12.0(11.0-13.0)** |
| Tail length, µm | 35.5 | 40.0 | 25.5 | 36 | 35 | **34.4(25.5-40.0)** |
